# Association of oxidative balance score with chronic kidney disease: NHANES 1999-2018

**DOI:** 10.3389/fendo.2024.1396465

**Published:** 2024-06-11

**Authors:** Haibin Wen, Xianhua Li, Jiangming Chen, Yi Li, Nailong Yang, Ning Tan

**Affiliations:** ^1^ Department of Nephrology, Jiangbin Hospital of Guangxi Zhuang Autonomous Region, Nanning, China; ^2^ Department of Endocrinology and Metabolism, The Affiliated Hospital of Qingdao University, Qingdao, China; ^3^ Traditional Chinese Medicine Department, Guilin Maternal and Child Health Hospital, Guilin, China; ^4^ General Surgery Day Ward, Department of General Surgery, The Third People’s Hospital of Chengdu and The Affiliated Hospital of Southwest Jiaotong University, Chengdu, China; ^5^ College of Basic Medical Sciences, Guilin Medical University, Guilin, China

**Keywords:** chronic kidney disease, oxidative balance score, antioxidants and prooxidants, oxidative stress, cross-sectional

## Abstract

**Background:**

The Oxidative Balance Score (OBS), which quantifies the balance between antioxidants and pro-oxidants influenced by diet and lifestyle, is crucial given oxidative stress’s significant role in Chronic Kidney Disease (CKD). This study aims to determine the association between OBS and CKD using data from the National Health and Nutrition Examination Survey (NHANES) 1999-2018.

**Methods:**

We analyzed data from the National Health and Nutrition Examination Survey (NHANES) spanning 1999 to 2018. OBS was constructed from a detailed array of 20 factors, including dietary nutrients and lifestyle behaviors. The relationship between OBS and CKD risk was evaluated using weighted logistic regression models, adjusted for potential confounders, with a generalized additive model (GAM) examining non-linear associations. Subgroup analyses and interaction effects across diverse demographic and clinical groups, along with sensitivity analyses, were performed to validate the findings.

**Results:**

Among 32,120 participants analyzed, 4,786 were identified with CKD. Fully adjusted weighted logistic regression analysis revealed that each unit increase in OBS was associated with a 2% reduction in CKD prevalence [OR: 0.98 (0.98–0.99), P < 0.001]. Higher OBS quartiles were significantly correlated with a decreased CKD risk [Q4 *vs*. Q1: OR: 0.82 (0.68–0.98), P = 0.03; P for trend = 0.01]. The GAM and smoothed curve fit indicated a linear relationship between OBS and the risk of CKD. Stratified and sensitivity analyses further substantiated the inverse relationship between OBS and CKD prevalence.

**Conclusions:**

Our findings from the NHANES data affirm a significant inverse association between OBS and CKD risk in the U.S. population, underscoring the role of optimizing dietary and lifestyle factors in managing CKD risk. These results advocate for incorporating OBS considerations into CKD prevention and treatment strategies.

## 1 Introduction

Chronic Kidney Disease (CKD), characterized by a reduced glomerular filtration rate [eGFR < 60 mL/min/1.73 m²) or the presence of albuminuria (urine albumin-creatinine ratio (UACR) ≥ 30 mg/g], represents a significant and escalating global health concern (1). According to the Global Burden of Disease (GBD), CKD ranks as the 12th leading cause of death among 133 diseases (2). In the United States alone, approximately 37 million adults are affected, markedly increasing their risk for cardiovascular diseases, hospitalizations, in-hospital complications, and premature mortality (3). This growing prevalence underscores the urgency for medical practitioners to prioritize CKD in their clinical practice, highlighting the need for innovative research into modifiable risk factors and preventive strategies.

Oxidative Balance Score (OBS), a comprehensive tool for assessing an individual’s balance between oxidative and antioxidant status, is derived by quantifying the antioxidant and pro-oxidant components present in dietary and lifestyle factors (4). An optimal balance is crucial for cellular and systemic health, as imbalances can contribute to the pathogenesis of various chronic conditions (5, 6). OBS has been correlated with several chronic diseases, including type 2 diabetes (7), non-alcoholic fatty liver disease (8), depression (9), cardiovascular disease (10), cancer (11), and impaired sleep quality (12).

Some preliminary studies have suggested a potential association between higher Oxidative Balance Scores (OBS) and a lower risk of Chronic Kidney Disease (CKD). For instance, an analysis based on the Korean Genome and Epidemiology Study found a significant association between higher OBS and lower CKD risk (13). Similarly, the Reasons for Geographic and Racial Differences in Stroke (REGARDS) cohort study reported comparable findings (14). However, these initial observations were primarily derived from single-population or small-scale samples, highlighting the need for more comprehensive epidemiological evidence from larger, multi-ethnic populations. Based on this backdrop, our study hypothesizes that a higher Oxidative Balance Score is associated with a lower risk of developing Chronic Kidney Disease. This hypothesis drives our utilization of data from the National Health and Nutrition Examination Survey (NHANES), aiming to evaluate the association between OBS and CKD risk among a nationally representative sample of the US population. Our study seeks to provide more reliable and generalizable evidence, potentially informing public health strategies and clinical practices in managing CKD.

## 2 Materials and methods

### 2.1 Study design and participants

The National Health and Nutrition Examination Survey (NHANES) is a series of cross-sectional surveys designed to assess the health and nutritional status of the non-institutionalized civilian population in the United States. This survey integrates demographic, socioeconomic, dietary, and health-related data collected through face-to-face interviews, physical examinations, and extensive laboratory tests. The comprehensive methodology and value of NHANES have been previously described in detail (15–17).

**Figure 1:**
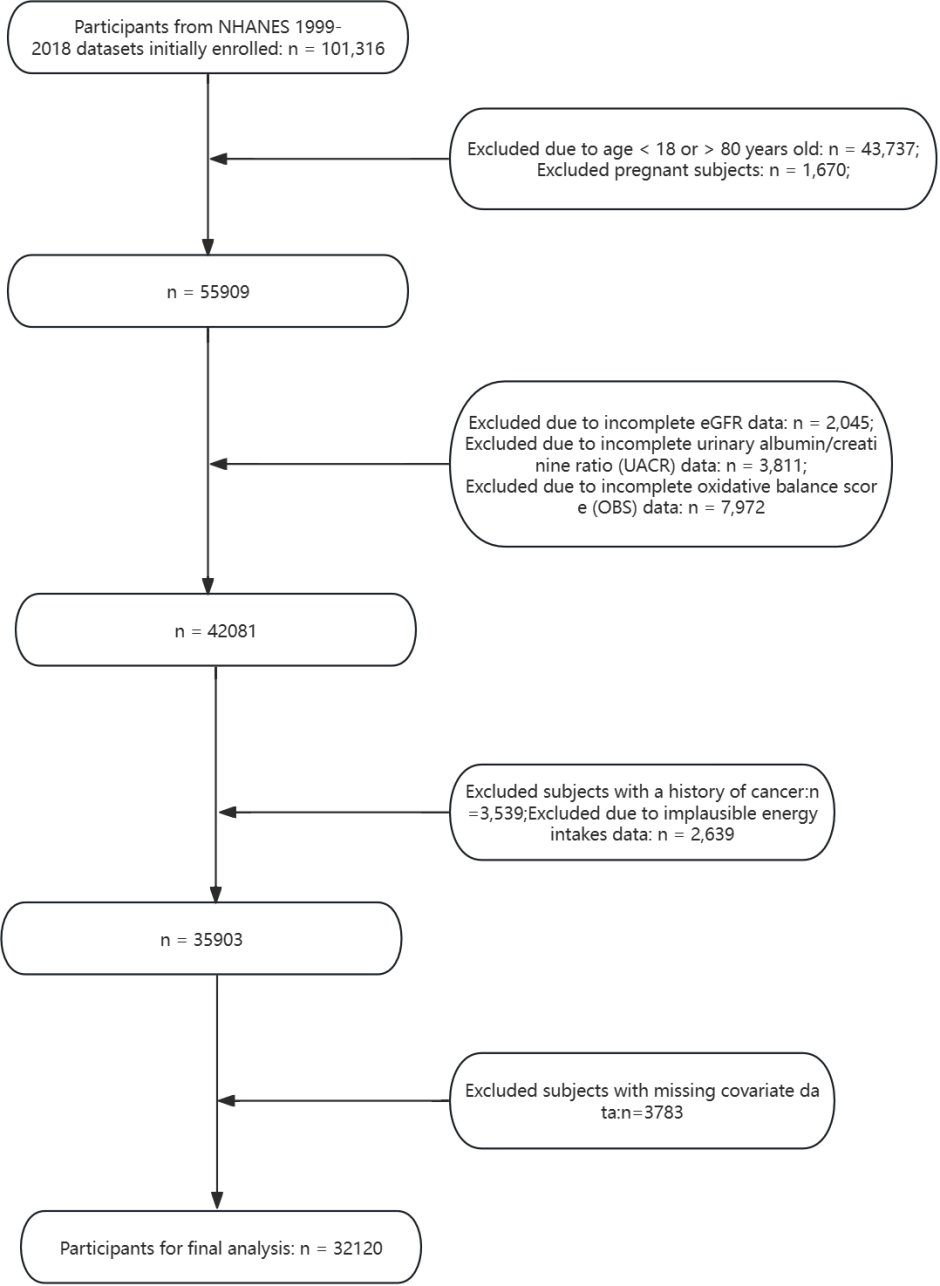
A flowchart showing the selection of study participants.

In our cross-sectional analysis of the National Health and Nutrition Examination Survey (NHANES) data from 1999 to 2018, we initially included 101,316 participants. Participants outside the age range of 18 to 80 years (43,737 individuals) were first excluded. Additional exclusions were made for pregnant subjects (1,670), those with incomplete data on key indicators including estimated glomerular filtration rate (eGFR) (2,045), urinary albumin/creatinine ratio (UACR) (3,811), and oxidative balance score (OBS) (7,972). We further excluded individuals with a history of cancer (3,539) and those with implausible energy intake data (2,639). The final step removed subjects with missing data on essential covariates (3,783), resulting in a study population of 32,120 participants (**Figure 1**).

### 2.2 Ethical considerations

National Health and Nutrition Examination Survey (NHANES) was conducted in accordance with the ethical standards laid down by the National Centre for Health Statistics’ Ethical Review Committee. Prior to participation, all participants were provided with comprehensive information regarding the study’s aims, procedures, potential risks, and benefits. Written informed consent was obtained from each participant, ensuring their voluntary participation and the confidentiality of their personal and health information.

### 2.3 Data collection

#### 2.3.1 Exposure variable: oxidative balance score

The Oxidative Balance Score (OBS) is a composite measure designed to quantify the balance between pro-oxidant and antioxidant exposures in the diet and lifestyle of individuals. The methodology for constructing and calculating OBS has been previously described in detail (18).

OBS construction incorporates a comprehensive array of 20 factors, encompassing both dietary nutrients and lifestyle behaviors. These factors are categorized into pro-oxidants, which include total fat, iron, alcohol intake, body mass index (BMI), and cotinine levels, and antioxidants, which comprise dietary fiber, β-carotene, vitamins B2, niacin, B6, total folate, B12, C, E, and minerals such as calcium, magnesium, zinc, copper, selenium, alongside physical activity. The assignment of scores to each component is based on its oxidative property (either antioxidant or prooxidant) and differentiated by gender, adhering to a predefined OBS component assignment scheme. Specifically, the OBS component assignment scheme incorporates alcohol consumption levels, categorizing participants into non-drinkers, non-heavy drinkers (0 to 15 g/day for women and 0 to 30 g/day for men), and heavy drinkers (≥15 g/day for women and ≥30 g/day for men). Scores of 2, 1, and 0 points are allocated to these categories, respectively. The scoring for other components is determined through tertiles based on gender, with antioxidant groups receiving scores from 0 to 2 points from the lowest to the highest tertile, whereas prooxidant groups are scored inversely, with the highest tertile receiving 0 points and the lowest tertile 2 points (18).

#### 2.3.2 Outcome variable: chronic kidney disease

The outcome variable of interest in this study is Chronic Kidney Disease (CKD), which is evaluated through two primary markers: estimated glomerular filtration rate (eGFR) and albuminuria. The eGFR is calculated using the Chronic Kidney Disease Epidemiology Collaboration (CKD-EPI) equation, which incorporates serum creatinine levels to assess kidney function (19). Albuminuria, is determined by the ratio of urine albumin to creatinine, providing an additional measure of kidney health. CKD is defined according to the current clinical guidelines as an eGFR of less than 60 mL/min/1.73 m², albuminuria of 30 mg/g or greater, or both conditions (20).

#### 2.3.3 Covariate definitions

The covariates were selected based on their potential impact on kidney health and included sociodemographic factors, lifestyle characteristics, and comorbid conditions.

##### 2.3.3.1 Sociodemographic characteristics

Participants’ demographic information was collected through a self-administered questionnaire, which included details on age, gender, and race/ethnicity. Race/ethnicity was classified into four categories: Mexican American, Non-Hispanic White, Non-Hispanic Black, and Other. Body Mass Index (BMI) was calculated using the formula: weight (kg)/height squared (m²), and categorized into three groups: underweight/normal (<25 kg/m²), overweight (25–30 kg/m²), and obese (≥30 kg/m²) (21). The poverty-to-income ratio, a measure of socioeconomic status, was determined by dividing the household income by the poverty threshold for the survey year and state, providing insight into the participants’ economic conditions.

##### 2.3.3.2 Lifestyle characteristics

Dietary intake was assessed using a 24-hour dietary recall interview, which captured detailed information on all food and beverage consumption from the previous day. This method provided a snapshot of the participant’s daily caloric intake, essential for evaluating dietary habits and their potential impact on health outcomes. Total energy intake was calculated in kilocalories per day (kcal/day) based on the data from the first dietary recall (22).

##### 2.3.3.3 Comorbidity

Hypertension was defined as having a systolic blood pressure (BP) ≥140 mmHg, diastolic BP ≥90 mmHg, or current use of antihypertensive medication (23).Diabetes and Prediabetes classifications adhered to the American Diabetes Association (ADA) criteria, utilizing fasting blood glucose (FBG) and Hemoglobin A1c (HbA1c) thresholds (24).Hyperlipidemia was identified through specific lipid profile levels or the use of lipid-lowering medications (25).

### 2.4 Statistical analysis

The study population was stratified into quartiles based on their Oxidative Balance Score (OBS) levels to facilitate comparative analysis. Continuous variables are presented as means ± standard errors (SE); Categorical variables were shown as frequency (%).

To evaluate the differences among the OBS quartiles for continuous variables, Analysis of Variance (ANOVA) was utilized. Subsequent *post-hoc* tests were conducted to identify specific group differences. For categorical variables, the Chi-square test or Fisher’s exact test was applied to examine differences across OBS quartiles, depending on the expected frequencies in the contingency tables.

OBS levels were analyzed both as a continuous variable (per unit increase) and in a categorized format (quartiles). The relationship between OBS levels and chronic kidney disease (CKD) prevalence was examined through weighted logistic regression analyses, with adjustments for various covariates. These analyses were structured in a hierarchical fashion, starting with a crude model and sequentially adjusting for sociodemographic factors, lifestyle characteristics, and comorbid conditions. To investigate potential non-linear associations between OBS and CKD, generalized additive models (GAMs) with smooth curve fittings were utilized, uncovering the complex relationship dynamics. Subgroup analyses and the assessment of interaction effects were performed using weighted logistic regression across different demographic and clinical categories. Sensitivity analyses were conducted independently for dietary and lifestyle Oxidative Balance Scores (OBS) to evaluate their respective associations with Chronic Kidney Disease (CKD) risk, utilizing weighted logistic regression models adjusted for potential confounders. All the analyses were performed with the statistical software packages R version 4.2.0 (http://www.R-project.org)), FreeStatistics software version 1.8, and EmpowerStats (http://www.empowerstats.com). P value <0.05 was considered statistically significant.

## 3 Results

In this study (**Table 1**), 32,120 participants were stratified into quartiles based on their Oxidative Balance Score (OBS).The demographics and health characteristics showed significant variations across quartiles. The gender distribution was notably different (P=0.001), with a higher percentage of males in the lower quartiles. Race also significantly varied, with Non-Hispanic Whites predominating in the highest OBS quartile (P<0.0001).Socioeconomic status, as indicated by the Poverty to Income Ratio (PIR), and dietary habits, reflected through energy intake, significantly differed across OBS levels (P<0.0001 for both).Key markers of kidney function, including the urine albumin to creatinine ratio (uACR), showed significant differences (P=0.002), while estimated glomerular filtration rate (eGFR) did not (P=0.13). The prevalence of diabetes, hypertension, and hyperlipidemia was significantly higher in lower OBS quartiles (P<0.0001), aligning with an increased CKD prevalence in these groups (P<0.0001). In **Supplementary Material 1**, we utilized box plots to depict the distribution of Oxidative Balance Scores (OBS) among groups with and without Chronic Kidney Disease. **Supplementary Material 2** presents the comparison of components of the Oxidative Balance Score (OBS) between CKD and Non-CKD Groups.

**Table 1:** Baseline Characteristics of participants based on OBS quartiles.

Variable	Total	Q1	Q2	Q3	Q4	Pvalue
Age (years)	45.17 (0.23)	45.05 (0.30)	45.38 (0.29)	44.88 (0.30)	45.31 (0.35)	0.42
Gender,n (%)						0.001
Male	15499 (47.61)	4396 (49.39)	4235 (49.20)	3473 (45.79)	3395 (46.32)	
Female	16621 (52.39)	4344 (50.61)	4426 (50.80)	3813 (54.21)	4038 (53.68)	
Race,n (%)						<0.0001
Non-Hispanic White	13859 (68.96)	3329 (62.85)	3606 (67.43)	3251 (69.05)	3673 (74.91)	
Non-Hispanic Black	6682 (10.54)	2597 (17.55)	1914 (11.61)	1260 (8.95)	911 (5.58)	
Mexican American	6181 (8.38)	1626 (7.84)	1729 (8.79)	1457 (9.25)	1369 (7.70)	
Other Race	5398 (12.12)	1188 (11.76)	1412 (12.17)	1318 (12.75)	1480 (11.81)	
PIR	3.04 (0.03)	2.53 (0.03)	2.93 (0.04)	3.14 (0.03)	3.45 (0.04)	<0.0001
Energy intake (kcal/d)	2101.28 (6.82)	1623.99 (9.80)	1985.76 (12.96)	2212.32 (12.95)	2474.23 (10.83)	<0.0001
CKD_EPI_Scr_2009	95.99 (0.30)	96.04 (0.41)	95.47 (0.38)	96.51 (0.40)	95.97 (0.43)	0.13
ACR (mg/g)	26.93 (1.23)	34.66 (2.95)	27.80 (2.30)	21.69 (1.89)	24.67 (2.46)	0.002
DM,n (%)						<0.0001
No	25108 (80.79)	6584 (77.88)	6679 (79.40)	5713 (80.48)	6132 (84.52)	
IGT	863 (3.01)	190 (2.49)	228 (3.13)	233 (3.28)	212 (3.05)	
IFG	1291 (4.14)	371 (4.20)	346 (4.24)	336 (4.83)	238 (3.44)	
DM	4858 (12.06)	1595 (15.43)	1408 (13.23)	1004 (11.41)	851 (8.99)	
Hypertension,n (%)						<0.0001
No	20200 (65.84)	5060 (60.90)	5329 (63.31)	4711 (66.83)	5100 (71.06)	
Yes	11920 (34.16)	3680 (39.10)	3332 (36.69)	2575 (33.17)	2333 (28.94)	
Hyperlipidemia,n (%)						<0.0001
No	9610 (30.53)	2326 (26.00)	2483 (28.76)	2207 (30.27)	2594 (35.78)	
Yes	22510 (69.47)	6414 (74.00)	6178 (71.24)	5079 (69.73)	4839 (64.22)	
CKD,n (%)						<0.0001
No	27334 (87.72)	7149 (84.79)	7262 (86.18)	6339 (88.91)	6584 (90.34)	
Yes	4786 (12.28)	1591 (15.21)	1399 (13.82)	947 (11.09)	849 (9.66)	

PIR, Poverty Income Ratio; DM, Diabetes Mellitus; IGT, Impaired Glucose Tolerance; IFG, Impaired Fasting Glucose.

Continuous Variables: Presented as means with standard errors (SE); Categorical Variables: Displayed as counts (n) and percentages (%).

As indicated in **Table 2**, in the unadjusted model, each incremental unit increase in OBS was correlated with a 3% decrease in the odds of developing CKD.(OR=0.97, 95% CI: 0.96-0.98, P<0.0001). This association remained significant across all adjusted models, with the fully adjusted model (Adjust III model) showing a 2% reduction per unit increase in OBS (OR=0.98, 95% CI: 0.98-0.99, P<0.001).

**Table 2:** Relationship between OBS and CKD in different models.

Exposure Variable	Non-adjusted model OR (95%CI) Pvalue	Adjust I model OR (95%CI) Pvalue	Adjust II model OR (95%CI) Pvalue	Adjust III model OR (95%CI) Pvalue
OBS(per unit change)	0.97 (0.96,0.98), <0.0001	0.98 (0.97,0.98), <0.0001	0.98 (0.97,-0.99), <0.0001	0.98 (0.98,0.99), <0.001
OBS quartile				
Q1	ref	ref	ref	ref
Q2	0.89 (0.80,1.00) 0.06	0.94 (0.83,1.06) 0.32	0.97 (0.84,1.10) 0.61	1.00 (0.87,1.14) 0.94
Q3	0.70 (0.62,0.79) <0.0001	0.77 (0.68,0.87) <0.0001	0.80 (0.70,0.93) 0.003	0.84 (0.73,0.98) 0.03
Q4	0.60 (0.52,0.68) <0.0001	0.68 (0.58,0.79) <0.0001	0.73 (0.61,0.87) <0.001	0.82 (0.68,0.98) 0.03
P for trend	<0.0001	<0.0001	<0.001	0.01

Non-adjusted model: adjusted for no covariates.

Model I: Adjustments are made for Age, Gender, Race, and Poverty-to-Income Ratio (PIR).

Model II: This model includes all adjustments from Model I, with additional adjustments for energy intake.

Model III: Extends the adjustments of Model II to include comorbidities: Hypertension, Diabetes, Hyperlipidemia.

When OBS was categorized into quartiles, the highest quartile (Q4) exhibited the strongest association with a lower risk of CKD compared to the reference first quartile (Q1), with a gradually decreasing trend in odds ratios (ORs) across the quartiles. Specifically, in the fully adjusted model, Q4 had an OR of 0.82 (95% CI: 0.68-0.98, P=0.03) compared to Q1, indicating a significant trend across OBS quartiles (P for trend=0.01).

Figure Annotation: This generalized additive model (GAM) plot illustrates the relationship between OBS concentration (X-axis) and the Risk of CKD (Y-axis). The red curve represents the fitted line, while the blue shaded area denotes the 95% confidence interval.

**Figure 2:**
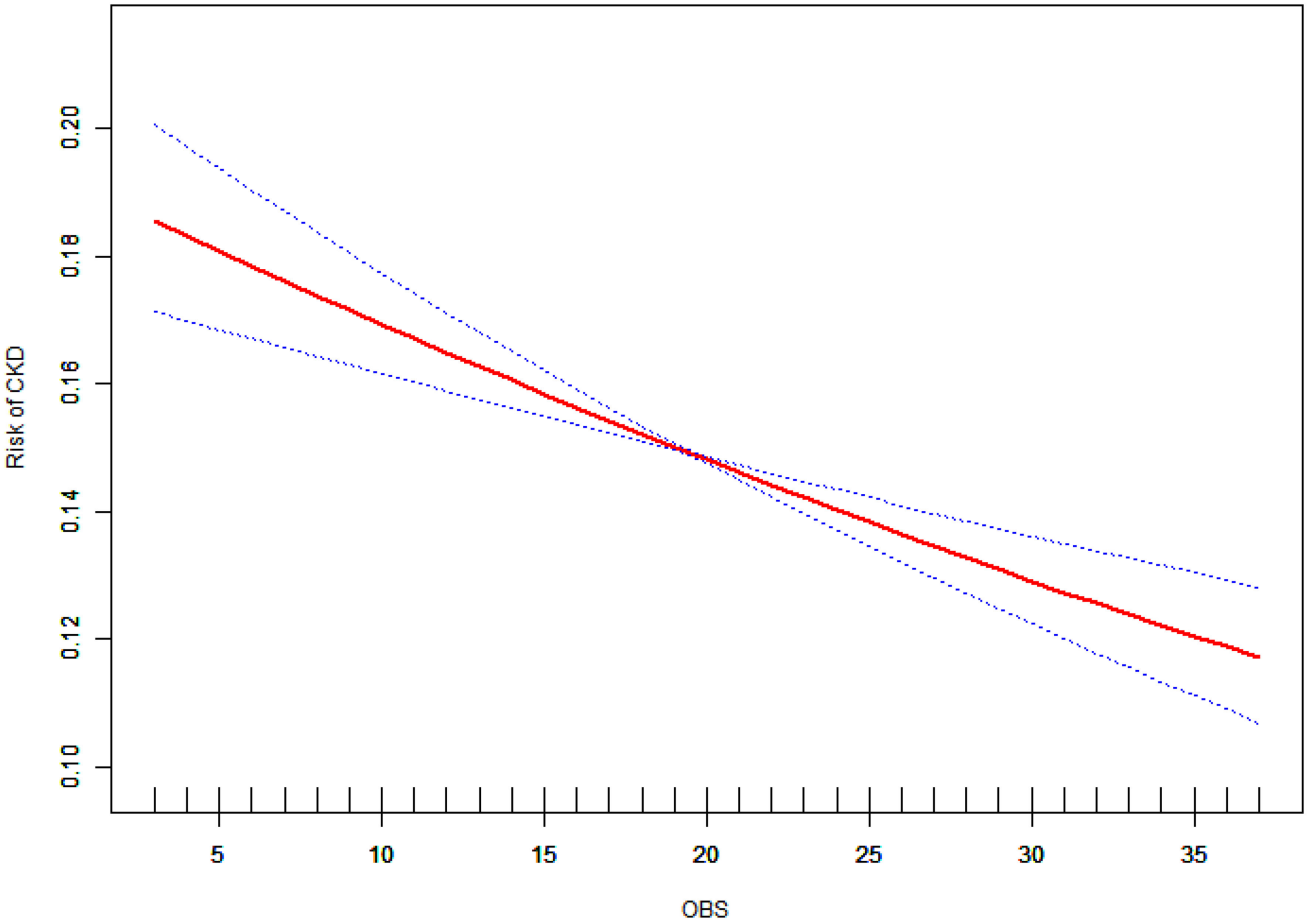
Smoothed Curve Fitting of Oxidative Balance Score and Risk of Chronic Kidney Disease.

**Figure 2** presents a curve fitting analysis using a generalized additive model (GAM) to elucidate the relationship between the Oxidative Balance Score (OBS) and chronic kidney disease (CKD). This analysis adjusts for a wide array of confounders, including demographic factors (age, gender, race, and poverty to income ratio - PIR), lifestyle factors (energy intake), and prevalent comorbid conditions (hypertension, diabetes mellitus, hyperlipidemia). The GAM-derived curve clearly demonstrates an inverse correlation between OBS and CKD, without significant inflection points.

In **Table 3**, the Oxidative Balance Score was categorized into four quartiles, designated as Q1, Q2, Q3, and Q4. Specifically, Q1 represents the lowest 25% of observed values, Q2 represents the 25th to 50th percentile of observed values, Q3 represents the 50th to 75th percentile of observed values, and Q4 represents the highest 25% of observed values. We conducted stratified analyses by age, sex, race, poverty income ratio, BMI, diabetes status, hypertension, and hyperlipidemia, and tested for potential interactions between these factors and OBS on CKD risk. Overall, the inverse association between higher OBS and lower CKD risk was consistently observed across strata, although the strength of this association appeared to vary by certain demographic and clinical factors. For instance, the inverse OBS-CKD relationship was most pronounced among adults aged >60 years, males, non-Hispanic Whites, those with higher income levels, and overweight individuals. This inverse association was attenuated in obese participants and those with diabetes. However, most interaction tests between OBS and the stratifying factors on CKD risk were not statistically significant, suggesting a generally consistent protective effect of higher OBS across the overall population.

**Table 3:** Stratified Analysis and Interaction Tests for the Association Between Oxidative Balance Score and Risk of Incident Chronic Kidney Disease in U.S. Adults: NHANES 1999–2018.

Character	Q1	Q2	Q3	Q4	p for interaction
**age**					0.066
<= 60 years	ref	1.137 (0.938,1.378)	0.884 (0.716,1.091)	0.945 (0.734,1.215)	
> 60 years	ref	0.755 (0.626,0.910)	0.744 (0.587,0.944)	0.647 (0.510,0.822)	
**Gender**					0.173
Male	ref	0.826 (0.677,1.007)	0.757 (0.614,0.933)	0.724 (0.555,0.945)	
Female	ref	1.160 (0.972,1.384)	0.926 (0.756,1.133)	0.910 (0.734,1.126)	
**Race**					0.387
Non-Hispanic White	ref	0.964 (0.793,1.170)	0.843 (0.678,1.049)	0.806 (0.632,1.027)	
Non-Hispanic Black	ref	1.097 (0.909,1.325)	0.967 (0.726,1.288)	0.872 (0.643,1.183)	
Mexican American	ref	0.883 (0.698,1.118)	0.824 (0.600,1.132)	0.926 (0.677,1.267)	
Other Race	ref	1.002 (0.694,1.446)	0.631 (0.435,0.915)	0.664 (0.421,1.047)	
**PIR**					0.282
Low (<1.3)	ref	1.020 (0.842,1.235)	0.800 (0.622,1.028)	1.000 (0.770,1.297)	
Medium (1.3-1.8)	ref	1.129 (0.820,1.554)	0.880 (0.618,1.253)	0.961 (0.642,1.438)	
High (>1.8)	ref	0.951 (0.790,1.145)	0.838 (0.690,1.018)	0.757 (0.597,0.959)	
**BMI**					0.337
Normal(<25)	ref	1.231 (0.965,1.571)	0.891 (0.667,1.191)	1.001 (0.731,1.370)	
Overweight(25 to <30)	ref	0.921 (0.711,1.193)	0.698 (0.541,0.900)	0.608 (0.450,0.821)	
Obese(30 or greater)	ref	0.937 (0.761,1.155)	0.926 (0.727,1.178)	0.858 (0.655,1.124)	
**DM**					0.275
no	ref	1.120 (0.949,1.321)	0.902 (0.749,1.087)	0.906 (0.727,1.130)	
IGT	ref	0.512 (0.229,1.146)	0.775 (0.395,1.520)	0.917 (0.386,2.176)	
IFG	ref	1.023 (0.577,1.816)	1.178 (0.667,2.081)	1.189 (0.546,2.593)	
DM	ref	0.814 (0.625,1.059)	0.679 (0.500,0.922)	0.561 (0.415,0.759)	
**Hypertension**					0.904
no	ref	1.066 (0.882,1.288)	0.899 (0.700,1.156)	0.903 (0.692,1.180)	
yes	ref	0.931 (0.795,1.089)	0.784 (0.653,0.942)	0.732 (0.594,0.902)	
**Hyperlipidemia**					0.293
no	ref	1.189 (0.881,1.605)	0.904 (0.677,1.208)	0.963 (0.704,1.318)	
yes	ref	0.950(0.815,1.106)	0.830 (0.703,0.979)	0.781 (0.647,0.942)	

the Oxidative Balance Score was categorized into four quartiles, designated as Q1, Q2, Q3, and Q4. Q1 ref denotes the lowest quartile of the OBS as the reference category.P for interaction was calculated using weighted multivariable logistic regression analysis adjusted for age, gender, race, poverty-to-income ratio (PIR), energy intake, and comorbidities including hypertension, diabetes, and hyperlipidemia, excluding the specific subgroup variable.

Sensitivity analyses were conducted independently for dietary and lifestyle Oxidative Balance Scores (OBS) to evaluate their respective associations with Chronic Kidney Disease (CKD) risk, utilizing weighted logistic regression models adjusted for age, gender, race, poverty-to-income ratio (PIR), energy intake, and hypertension, diabetes, hyperlipidemia. Fully adjusted weighted logistic regression analysis indicated that each unit increase in dietary OBS was associated with a 2% reduction in CKD prevalence [OR: 0.98 (95% CI: 0.98–0.99), P = 0.002]. Similarly, for lifestyle OBS, each unit increase was linked to a 4% reduction in CKD prevalence [OR: 0.96 (95% CI: 0.92–0.99), P = 0.01] (**Supplementary Material 3**).

## 4 Discussion

This study provides a detailed exploration of the relationship between the Oxidative Balance Score (OBS) and the risk of Chronic Kidney Disease (CKD), utilizing data from the National Health and Nutrition Examination Survey (NHANES) 1999-2018. After adjusting for a wide range of covariates, our analysis revealed a significant inverse association between OBS and CKD. The demonstrated negative correlation between OBS and CKD risk highlights the importance of antioxidant and pro-oxidant balance in renal health. It opens the door to innovative preventive strategies focused on enhancing oxidative balance, possibly through dietary and lifestyle modifications. This approach could offer a novel pathway for CKD risk management, emphasizing the need for further research to elucidate the mechanisms through which OBS influences CKD development and progression.

The Oxidative Balance Score (OBS) employed in our study is a comprehensive composite measure comprising 20 factors representing dietary and lifestyle pro-oxidants and antioxidants, which has been extensively validated across multiple studies (26–29). The pro-oxidant factors (total fat, iron, alcohol, BMI, cotinine) are well-established contributors to oxidative stress through mechanisms such as free radical generation, lipid peroxidation, inflammatory processes, and endogenous oxidative burden. Antioxidant factors encompass dietary fiber (binds pro-oxidants), vitamins C/E (direct free radical scavengers), B vitamins (enzyme cofactors for antioxidant synthesis/repair), minerals like zinc/selenium (essential for antioxidant enzymes), and physical activity (enhances antioxidant defenses) (30).This comprehensive panel encapsulates the current understanding of key exposures influencing the oxidant-antioxidant balance, a critical determinant of chronic disease risk. While the OBS composition may evolve with new discoveries, the present selection represents a reliable theoretical framework substantiated by a robust evidence base. By integrating these 20 factors, OBS provides an integrated assessment of an individual’s oxidative status, offering a biologically plausible basis for interpreting our study findings and warranting further validation in larger cohorts.

Hypertension and dyslipidemia are important risk factors for chronic kidney disease (CKD) (31, 32). Therefore, investigating the association between the oxidative balance score (OBS) and these two conditions may help elucidate the potential mechanisms by which OBS could further influence the development and progression of CKD through modulating these common risk factors. A study based on the Korean Genome and Epidemiology Study found that higher OBS scores were significantly associated with a lower risk of incident hypertension (33). Although no direct research has examined the relationship between OBS and dyslipidemia, an analysis of NHANES 2011-2018 data revealed a significant negative correlation between OBS scores and the risk of metabolic syndrome, of which dyslipidemia is a core component, suggesting a potential association between OBS and lower dyslipidemia risk (34). Mechanistically, existing studies have demonstrated that antioxidants can lower blood pressure by enhancing the bioavailability of nitric oxide, improving endothelial function, and regulating the renin-angiotensin system (35–37). Furthermore, antioxidants may also reduce dyslipidemia through mechanisms such as decreasing oxidative modifications of cholesterol in circulation and modulating the activity of lipoprotein metabolism enzymes (38, 39). Although further research is needed for confirmation, these findings provide a biological basis for our hypothesis that OBS may influence the risks of hypertension and dyslipidemia. In summary, current evidence supports the notion that OBS could potentially affect the development and progression of CKD by modulating common risk factors such as blood pressure and lipid levels. This lays the foundation for future research on the potential applications of OBS in the prevention and management of CKD and its complications.

The Oxidative Balance Score (OBS), which quantitatively assesses an individual’s exposure to both antioxidants and pro-oxidants, emerges as a crucial metric for evaluating oxidative stress levels (27). The pivotal role of oxidative stress in the progression of CKD has been discussed in previous studies (40–44). Oxidative stress, instigates cellular damage through lipid peroxidation, DNA damage, and protein oxidation (45). Such damage initiates inflammatory responses and fibrotic processes, which are fundamental in CKD’s pathogenesis (46). Notably, the NF-κB pathway, integral to inflammation, and the TGF-β/Smad pathway, associated with fibrosis, represent critical signaling pathways through which oxidative stress impacts renal health detrimentally (47–49), emphasizing the potential of OBS in guiding both the understanding and management of CKD.

Consistent with findings from the Korean Genome and Epidemiology Study, our study demonstrates that higher Oxidative Balance Scores (OBS) are associated with a reduced risk of Chronic Kidney Disease (CKD), underscoring the potential benefits of dietary and lifestyle modifications (13). Extending these insights to an American demographic, our research affirms the significance of OBS in reducing CKD risk across diverse populations. Our research is distinguished by analyzing OBS’s direct impact on CKD risk within an American context, where diverse racial and socioeconomic factors may uniquely influence oxidative stress. Meanwhile, this conclusion is supported by the Reasons for Geographic and Racial Differences in Stroke (REGARDS) cohort study, which also indicated OBS’s protective role against CKD (14). Unlike the REGARDS study, our methodology incorporates a broader set of antioxidants and pro-oxidants, offering a more comprehensive measure of oxidative balance and potentially a more accurate reflection of its impact on renal health. Additionally, our analysis includes a wider age range, enhancing the representativeness of our findings across the national landscape. Furthermore, discrepancies in OBS calculation methods between studies highlight the need for standardization to enable more effective comparison and replication of findings. Our approach, which includes a wider variety of biochemical markers, could serve as a step toward this standardization, providing a foundation for future research and leading to more consistent and reliable measures of oxidative balance.

The primary strength of our study lies in leveraging a large, nationally representative dataset alongside a rigorous methodological framework. This framework encompasses validated data collection methods, comprehensive analytical strategies, and the innovative employment of a generalized additive model (GAM) plot to elucidate the relationship between the Oxidative Balance Score (OBS) and Chronic Kidney Disease (CKD) risk. Furthermore, our subgroup and sensitivity analyses further validate the stability of our study conclusions.

However, our study’s cross-sectional design, relying on the National Health and Nutrition Examination Survey (NHANES) data, limits our ability to infer causality between OBS levels and CKD. This limitation highlights the imperative for longitudinal research to more definitively ascertain the dynamics of this relationship (50).

Additionally, the limitations of this study include the use of self-reported dietary data, which is susceptible to recall and reporting biases that could compromise the accuracy of Oxidative Balance Score (OBS) calculations and the observed associations. To mitigate this measurement errors and improve estimates of usual intake, we applied the National Cancer Institute method (50). Furthermore, although we adjusted for a wide range of known confounders, the potential for residual confounding remains due to unmeasured or inadequately measured variables. These factors may affect the observed relationship between OBS and CKD risk, warranting cautious interpretation of our findings.

## 5 Conclusion

In summary, our examination of NHANES data demonstrates a significant inverse relationship between Oxidative Balance Score (OBS) and Chronic Kidney Disease (CKD) risk. This relationship underscores the importance of oxidative balance in the context of CKD and suggests the potential for dietary and lifestyle modifications to influence CKD risk. The cross-sectional nature of our study calls for further longitudinal investigations to explore these associations in depth and to evaluate the effectiveness of specific interventions.

## Data availability statement

Publicly available datasets were analyzed in this study. This data can be found here: https://www.cdc.gov/nchs/nhanes/index.htm.

## Ethics statement

The studies involving humans were approved by National Center for Health Statistics Ethics Review Board. The studies were conducted in accordance with the local legislation and institutional requirements. Written informed consent for participation was not required from the participants or the participants’ legal guardians/next of kin in accordance with the national legislation and institutional requirements.

## Author contributions

HW: Writing – review & editing, Writing – original draft, Software, Formal analysis, Data curation. XL: Writing – review & editing, Writing – original draft, Investigation, Formal analysis. JC: Writing – review & editing, Writing – original draft, Formal analysis, Data curation. YL: Writing – review & editing, Writing – original draft, Supervision, Investigation. NY: Writing – review & editing, Writing – original draft, Supervision, Funding acquisition. NT: Writing – review & editing, Writing – original draft, Visualization, Methodology, Conceptualization.
