# Supplementary material for: Association of oxidative balance score with chronic kidney disease: NHANES 1999-2018
Source: Front Endocrinol (Lausanne). 2024 Jun 11;15:1396465. doi: 10.3389/fendo.2024.1396465 (PMC11198875; doi:10.3389/fendo.2024.1396465)

Supplementary Material 1: Oxidative Balance Score (OBS) Distribution in Chronic Kidney Disease Cases and Non-Cases: A Box Plot Comparison


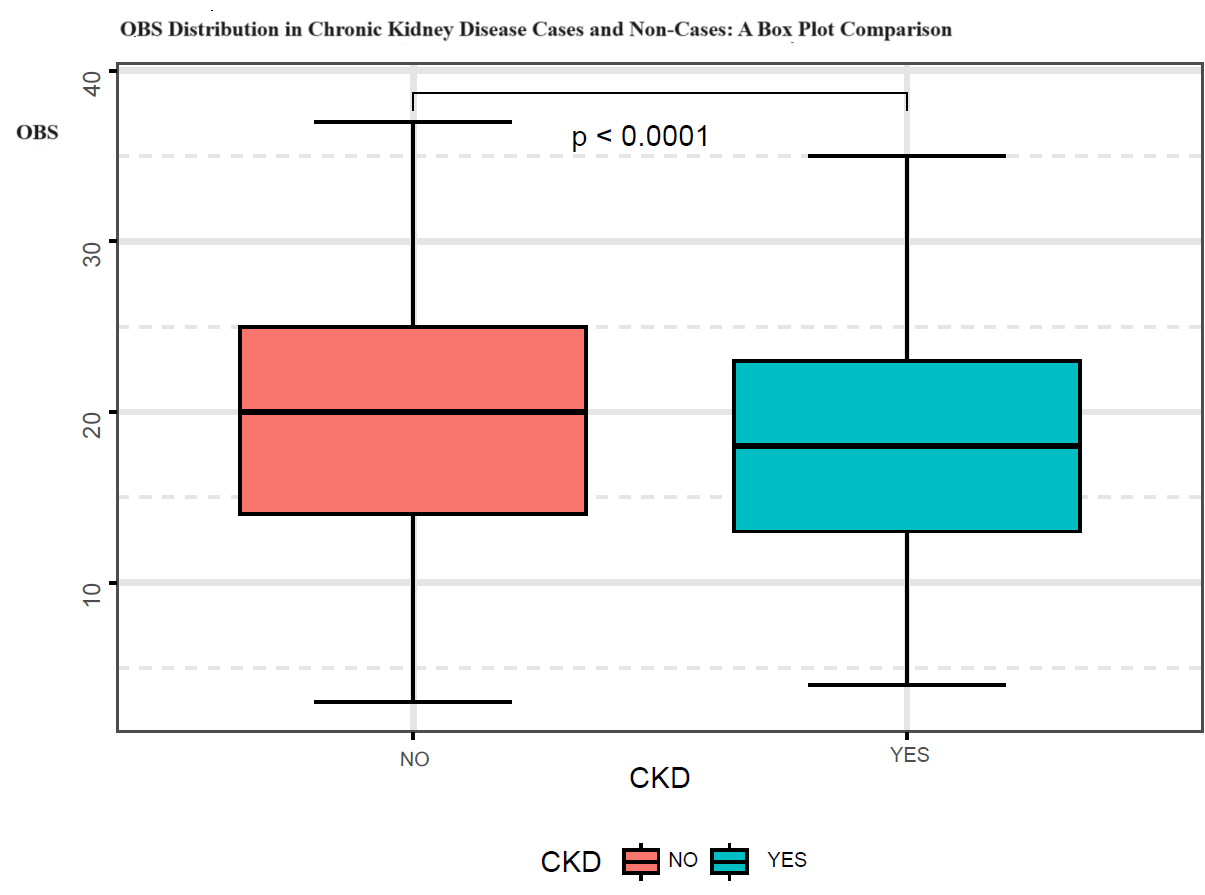

Supplement: Supplementary file 1 [file DataSheet_1.docx]
