# Supplementary material for: Association of oxidative balance score with chronic kidney disease: NHANES 1999-2018
Source: Front Endocrinol (Lausanne). 2024 Jun 11;15:1396465. doi: 10.3389/fendo.2024.1396465 (PMC11198875; doi:10.3389/fendo.2024.1396465)
Supplement: Supplementary file 2 [file DataSheet_2.docx]

Supplemental Table 2 Comparison of Components of the Oxidative Balance Score (OBS) between CKD and Non-CKD Groups

| variable | non-CKD | CKD | Pvalue |
| --- | --- | --- | --- |
| Dietary fiber(g/d) | 16.63(0.12) | 15.43(0.19) | < 0.0001 |
| Total fat (g/d) | 80.11(0.36) | 72.92(0.72) | < 0.0001 |
| Vitamin B2 (mg/d) | 2.16(0.01) | 1.97(0.02) | < 0.0001 |
| Niacin (mg/d) | 25.54(0.11) | 22.61(0.22) | < 0.0001 |
| Vitamin B6 (mg/d) | 2.07(0.01) | 1.86(0.02) | < 0.0001 |
| Total folate (mcg/d) | 403.44(2.40) | 366.98(4.79) | < 0.0001 |
| Vitamin B12 (mcg/d) | 5.14(0.05) | 4.80(0.10) | 0.003 |
| Vitamin C (mg/d) | 81.42(0.88) | 79.13(1.38) | 0.09 |
| Vitamin E (ATE) (mg/d) | 8.21(0.07) | 7.35(0.11) | < 0.0001 |
| Calcium (mg/d) | 942.81( 5.36) | 846.45(10.19) | < 0.0001 |
| Magnesium (mg/d) | 296.03(1.65) | 269.04(2.85) | < 0.0001 |
| Iron (mg/d) | 15.05(0.08) | 13.94(0.16) | < 0.0001 |
| Carotene (RE/d) | 204.69(3.39) | 199.40(5.70) | 0.39 |
| Zinc (mg/d) | 11.60(0.07) | 10.55(0.13) | < 0.0001 |
| Copper (mg/d) | 1.27(0.01) | 1.18(0.02) | < 0.0001 |
| Selenium (mcg/d) | 112.46(0.45) | 102.75(1.16) | < 0.0001 |
| Physical activity (MET-minute/week) | 3789.65( 84.51) | 3043.66(130.79) | < 0.0001 |
| Alcohol (g/d) | 8.99(0.23) | 7.43(0.50) | 0.005 |
| BMI ((kg/m^2^ ) | 28.65(0.08) | 30.20(0.16) | < 0.0001 |
| Cotinine (ng/ml) | 55.80(1.70) | 58.30(3.13) | 0.41 |

Continuous Variables: Presented as means with standard errors (SE)
