# Supplementary material for: Association of oxidative balance score with chronic kidney disease: NHANES 1999-2018
Source: Front Endocrinol (Lausanne). 2024 Jun 11;15:1396465. doi: 10.3389/fendo.2024.1396465 (PMC11198875; doi:10.3389/fendo.2024.1396465)
Supplement: Supplementary file 3 [file DataSheet_3.docx]

Supplemental Material 3:Associations Between Dietary and Lifestyle Oxidative Balance Scores and Chronic Kidney Disease Prevalence

| Exposure Variable | Non-adjusted model  OR(95%CI)  Pvalue | Adjust I model  OR(95%CI)  Pvalue | Adjust II model  OR(95%CI)  Pvalue | Adjust III model  OR(95%CI)  Pvalue |
| --- | --- | --- | --- | --- |
| Dietary OBS(per unit change) | 0.97 (0.97,0.98)，<0.0001 | 0.98(0.97,0.99)，<0.0001 | 0.98(0.97,0.99)，<0.001 | 0.98(0.98,0.99)，0.002 |
| Lifestyle OBS(per unit change) | 0.87 (0.84,0.90)，<0.0001 | 0.90(0.87,0.93)，<0.0001 | 0.90(0.87,0.93)，<0.0001 | 0.96(0.92,0.99)，0.01 |

Note:

Non-adjusted model: This model presents the basic relationship between OBS and CKD.

Model I: Adjustments are made for Age, Gender, Race, and Poverty-to-Income Ratio (PIR).

Model II: This model includes all adjustments from Model I, with additional adjustments for energy intake.

Model III: Extends the adjustments of Model II to include comorbidities: Hypertension, Diabetes, Hyperlipidemia.
